# Supplementary material for: Alternative TSS use is widespread in Cryptococcus fungi in response to environmental cues and regulated genome-wide by the transcription factor Tur1
Source: PLoS Biol. 2024 Jul 25;22(7):e3002724. doi: 10.1371/journal.pbio.3002724 (PMC11302930; doi:10.1371/journal.pbio.3002724)
Supplement: S1 Fig — (DOCX) [file pbio.3002724.s012.docx]

**Supplementary Figure S1 :** **TSS cluster sizes obtained after directly merging the twelve gff files originally generated in *Wallace et al* (14).**

(14) Wallace EWJ, Maufrais C, Sales-Lee J, Tuck LR, de Oliveira L, Feuerbach F, et al. Quantitative global studies reveal differential translational control by start codon context across the fungal kingdom. Nucleic Acids Res. 2020;48:2312-31.
