# Supplementary material for: Alternative TSS use is widespread in Cryptococcus fungi in response to environmental cues and regulated genome-wide by the transcription factor Tur1
Source: PLoS Biol. 2024 Jul 25;22(7):e3002724. doi: 10.1371/journal.pbio.3002724 (PMC11302930; doi:10.1371/journal.pbio.3002724)
Supplement: S2 Fig — (A) Stationary phase 30°C (B) exponential phase 37°C, and (C) stationary phase 37°C. Left. Distribution of the TSS clusters in terms of cluster width (size) and shape index (SI) is represented as a 2D density plot in which size and SI distribution of TSS clusters display a bimodal pattern. Corresponding histogram of size and SI are projected on x-axis and y-axis, respectively. Right. Histogram (bold blue line) and density plot (bold black line) of the first principal component (PC1) of size and SI. Two subpopulations of PC1 are detected by statistical test using “MixtureInf” R package and represented as the theoretical density plot (dotted black line). The data underlying this figure can be found in S1 Data. (DOCX) [file pbio.3002724.s013.docx]

**Supplementary Figure S2. TSS clusterization using *C. neoformans* TSS-seq data obtained in three other growth conditions also revealed two types of TSS (broad and sharp).** (A) Stationary phase 30°C (B) exponential phase 37°C, and (C) stationary phase 37°C**.** Left. Distribution of the TSS clusters in terms of cluster width (Size) and shape index (SI) is represented as a 2D density plot in which Size and SI distribution of TSS clusters display a bimodal pattern. Corresponding histogram of Size and SI are projected on x‑axis and y‑axis, respectively. Right. Histogram (bold blue line) and density plot (bold black line) of the first principal component (PC1) of Size and SI. Two subpopulations of PC1 are detected by statistical test using “MixtureInf” R package and represented as the theoretical density plot (dotted black line).
