# Supplementary material for: Alternative TSS use is widespread in Cryptococcus fungi in response to environmental cues and regulated genome-wide by the transcription factor Tur1
Source: PLoS Biol. 2024 Jul 25;22(7):e3002724. doi: 10.1371/journal.pbio.3002724 (PMC11302930; doi:10.1371/journal.pbio.3002724)
Supplement: S3 Fig — Enrichments and positions relative to the major position within the TSS cluster of 6 detected motifs within the TL sequence. (DOCX) [file pbio.3002724.s014.docx]

**Supplementary Figure S3. Similar motif enrichment was observed associated with broad and sharp clusters generated from each set of TSS-seq data**. Enrichments and positions relative to the major position within the TSS cluster of 6 detected motifs within the TL sequence.
