# Supplementary material for: Alternative TSS use is widespread in Cryptococcus fungi in response to environmental cues and regulated genome-wide by the transcription factor Tur1
Source: PLoS Biol. 2024 Jul 25;22(7):e3002724. doi: 10.1371/journal.pbio.3002724 (PMC11302930; doi:10.1371/journal.pbio.3002724)
Supplement: S4 Fig — The number of cells with Mea102 nuclear localization is indicated. (DOCX) [file pbio.3002724.s015.docx]

**Supplementary figure S4. Mae102 localization under exponential phase.** The number of cells with Mea102 nuclear localization is indicated
