# Supplementary material for: Alternative TSS use is widespread in Cryptococcus fungi in response to environmental cues and regulated genome-wide by the transcription factor Tur1
Source: PLoS Biol. 2024 Jul 25;22(7):e3002724. doi: 10.1371/journal.pbio.3002724 (PMC11302930; doi:10.1371/journal.pbio.3002724)
Supplement: S5 Fig — IGV visualization of RNA-seq (upper tracks) and TSS-seq (bottom tracks) at the CNAG_01019 (SOD1) and CNAG_04388 (SOD2) loci when C. neoformans cells were cultivated at 30°C under exponential or stationary phase, respectively. (DOCX) [file pbio.3002724.s016.docx]

**Supplementary Figure S5. altTSS usage regulation of *SOD1* and *SOD2* genes in exponential and stationary phases.** IGV visualization of RNA-seq (upper tracks) and TSS-seq (bottom tracks) at the CNAG_01019 (*SOD1*) and CNAG_04388 (*SOD2*) loci when *C. neoformans* cells were cultivated at 30°C under exponential or stationary phase, respectively.
