# Supplementary material for: Alternative TSS use is widespread in Cryptococcus fungi in response to environmental cues and regulated genome-wide by the transcription factor Tur1
Source: PLoS Biol. 2024 Jul 25;22(7):e3002724. doi: 10.1371/journal.pbio.3002724 (PMC11302930; doi:10.1371/journal.pbio.3002724)
Supplement: S6 Fig — IGV visualization of RNA-seq and TSS-seq at the VPS70 and PKP1 loci of C. neoformans and C. deneoformans obtained when cells were cultivated at 30°C under stationary phase. At the C. deneoformans VPS70 gene, the same altTSS observed in C. neoformans is visible albeit very poorly used. At the C. deneoformans PKP1 gene, an altTSS is also visible but not at the same position as the one observed in C. neoformans. Interestingly, in both cases, the regulation of altTSS is reversed between species. (DOCX) [file pbio.3002724.s017.docx]

**Supplementary Figure S6. Apparent “non-conserved” altTSS can be conserved between *C. neoformans* and *C. deneoformans*.** IGV visualization of RNA-seq and TSS-seq at the *VPS70* and *PKP1* loci of *C. neoformans* and *C. deneoformans* obtained when cells were cultivated at 30°C under stationary phase. At the *C. deneoformans* *VPS70* gene, the same altTSS observed in *C. neoformans* is visible albeit very poorly used. At the *C. deneoformans* *PKP1* gene, an altTSS is also visible but not at the same position as the one observed in *C. neoformans*. Interestingly, in both cases, the regulation of altTSS is reversed between species.
