# Supplementary material for: Alternative TSS use is widespread in Cryptococcus fungi in response to environmental cues and regulated genome-wide by the transcription factor Tur1
Source: PLoS Biol. 2024 Jul 25;22(7):e3002724. doi: 10.1371/journal.pbio.3002724 (PMC11302930; doi:10.1371/journal.pbio.3002724)
Supplement: S7 Fig — Ten-fold serial dilutions (starting with 107 cells/ml) of the wild type (WT), tur1 mutant (tur1Δ), and complemented strain (tur1Δ TUR1) cells were spotted on YPD medium containing Cumen hydroperoxide (CHP), NaNO2, sodium dithiocarbamate, rotenone, dimethyl malonate, or oligomycin. Plates were incubated 3 days at 30°C before being photographed. (DOCX) [file pbio.3002724.s018.docx]

**Supplementary Figure S7. Sensitivity of the *tur1Δ* mutant to oxidative agents.** 10-fold serial dilutions (starting with 10^7^ cells/mL) of the wildtype (WT), *tur1* mutant (*tur1Δ)* and complemented strain *(tur1Δ TUR1)* cells were spotted on YPD medium containing Cumen hydroperoxide (CHP), NaNO_2_, Sodium dithiocarbamate, rotenone, Dimethyl malonate, or oligomycin. Plates were incubated 3 days at 30°C before being photographed
