# Supplementary material for: Alternative TSS use is widespread in Cryptococcus fungi in response to environmental cues and regulated genome-wide by the transcription factor Tur1
Source: PLoS Biol. 2024 Jul 25;22(7):e3002724. doi: 10.1371/journal.pbio.3002724 (PMC11302930; doi:10.1371/journal.pbio.3002724)
Supplement: S8 Fig — (A) Left panel: Cumulative percentage of clusters that reach size when testing a range of d values from 1 to 50. Right panel: Correlation between d and percentage of clusters that reach a maximum size. The red rectangle illustrates the areas where d runs from 12 to 18 in both panels. (B) Left panel: The data in (A) is modelized as the red smooth curve. Right panel: Correlation between d and modelized percentage of clusters that reach maximum size, which reaches the maximum value at d = 17 (red line). The data underlying this figure can be found in S1 Data. (DOCX) [file pbio.3002724.s019.docx]

**Supplementary Figure S8. Optimization of TSS data clusterization.** (A) Left panel: Cumulative percentage of clusters that reach size when testing a range of d values from 1 to 50. Right panel: Correlation between d and percentage of clusters that reach a maximum size. The red rectangle illustrates the areas where d runs from 12 to 18 in both panels. (B) Left panel: The data in (A) is modelized as the red smooth curve. Right panel: Correlation between d and modelized percentage of clusters that reach maximum size, which reaches the maximum value at d=17 (red line).
