# Supplementary figures and images for: Alternative TSS use is widespread in Cryptococcus fungi in response to environmental cues and regulated genome-wide by the transcription factor Tur1
Source: PLoS Biol. 2024 Jul 25;22(7):e3002724. doi: 10.1371/journal.pbio.3002724 (PMC11302930; doi:10.1371/journal.pbio.3002724)

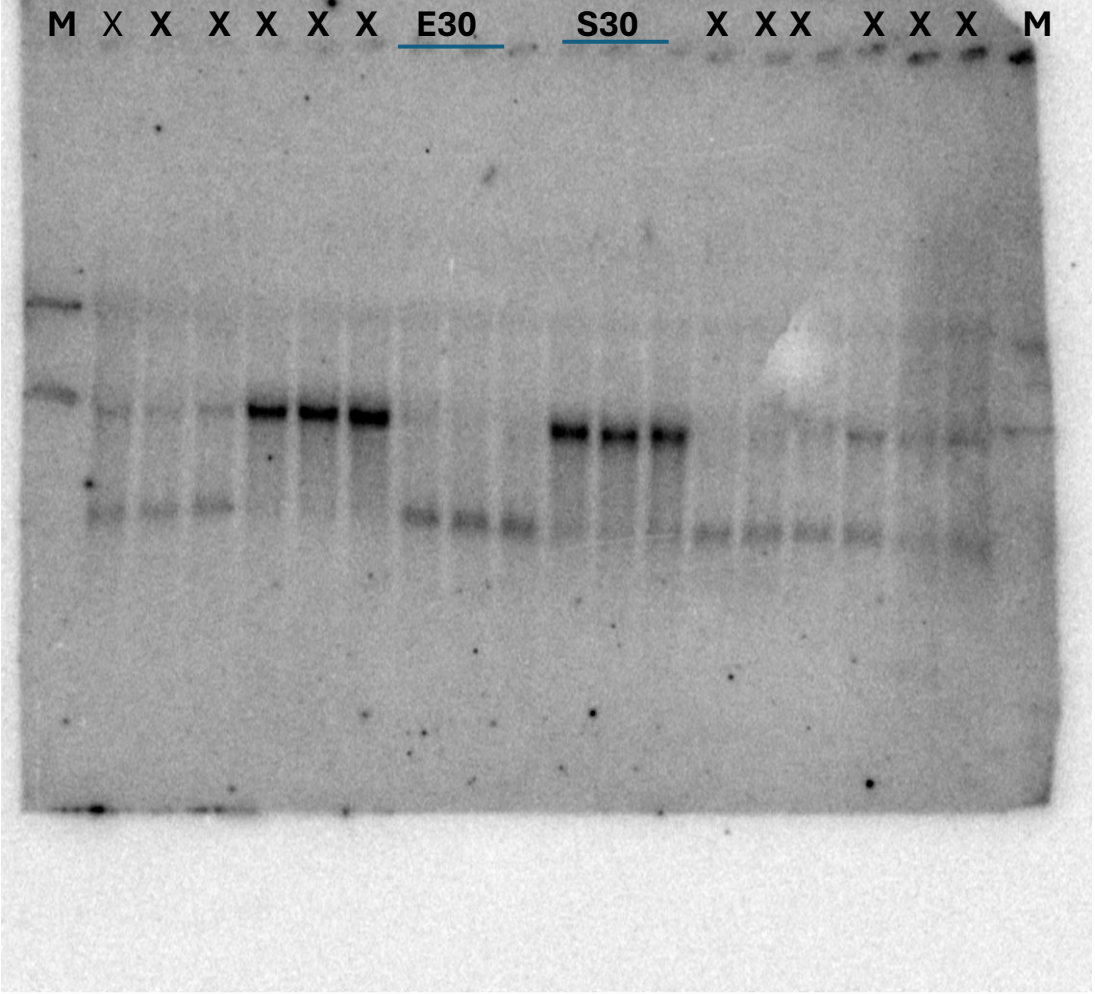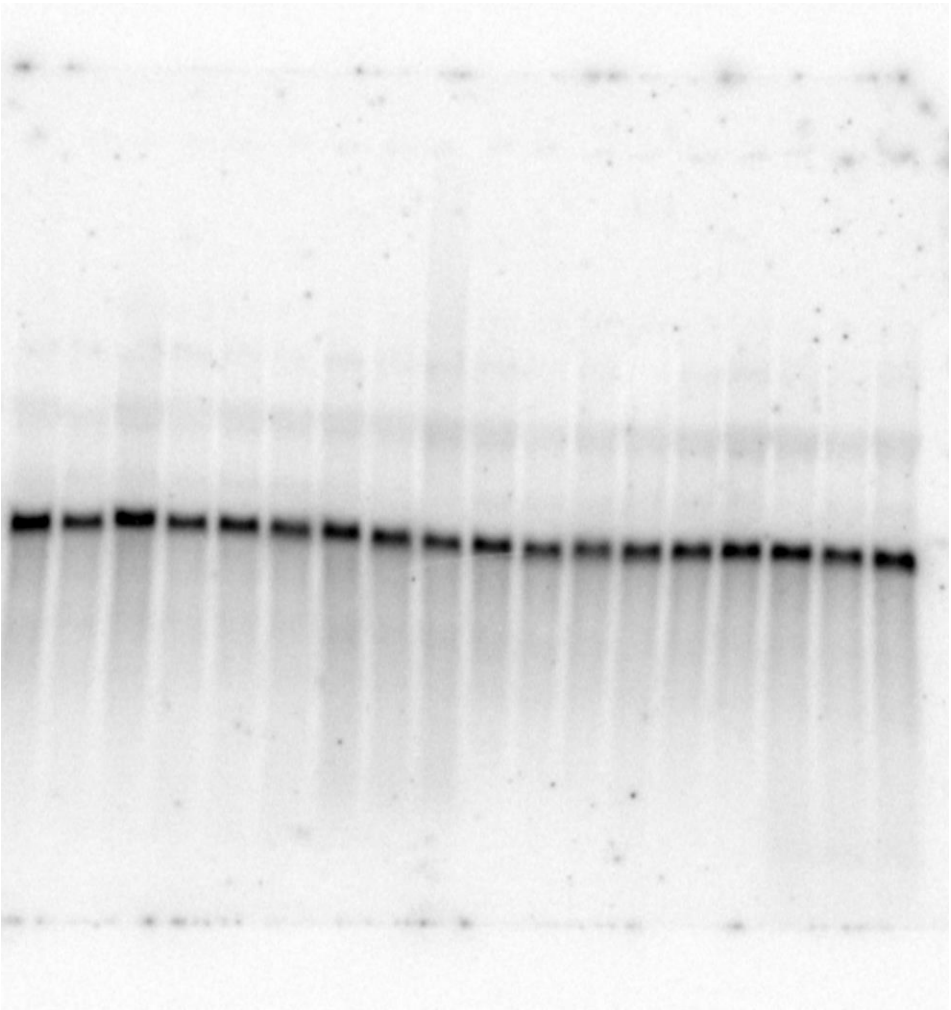

Figure 6 B

*P<sub>GAL7</sub>::FLAG2X-CBP::TUR1*

WT

YPD

YPGAL

YPD

YPGAL

E S

E S

E S

E S

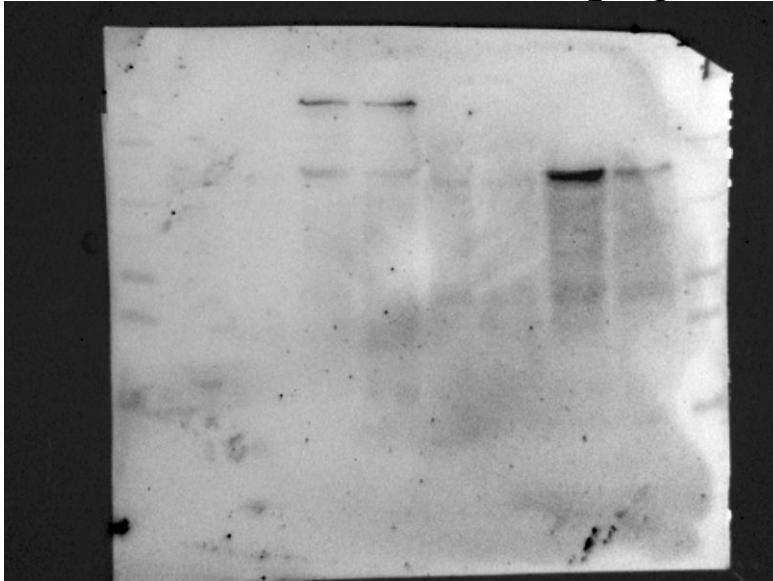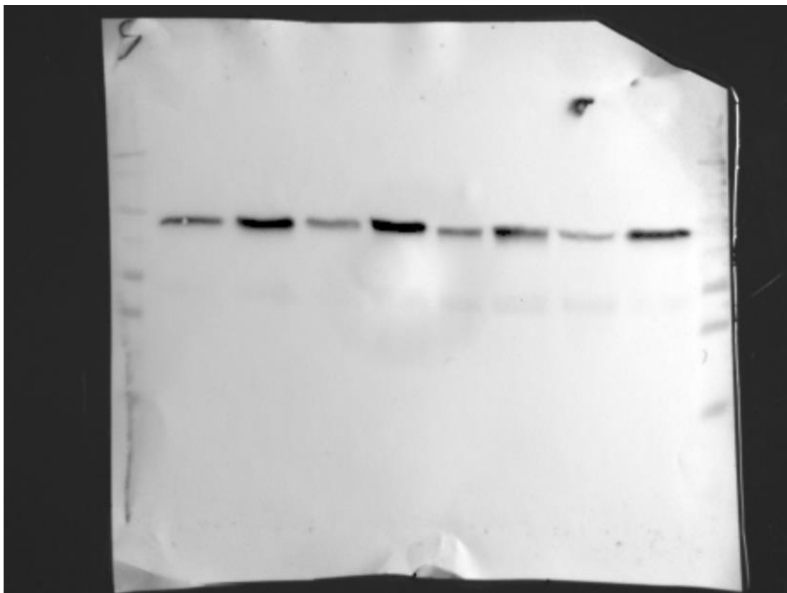

Figure 8C

Supplement: S1 Raw Images — (PDF) [file pbio.3002724.s020.pdf]
