# Supplementary material for: Alternative TSS use is widespread in Cryptococcus fungi in response to environmental cues and regulated genome-wide by the transcription factor Tur1
Source: PLoS Biol. 2024 Jul 25;22(7):e3002724. doi: 10.1371/journal.pbio.3002724 (PMC11302930; doi:10.1371/journal.pbio.3002724)
Supplement: Attachment — Submitted filename: Vi DANG et al.pdf [file pbio.3002724.s022.pdf]

The manuscript by Vi DANG et al. titled "Tur1 regulates alternative TSS usage in *Cryptococcus"* describes a new analysis of TSS data in *Cryptococcus* along with follow up experiments implicating the transcription factor Tur1 as a master regulator of aTSS. They demonstrate that many genes in *Cryptococcus* exhibit aTSS under different growth conditions, and that the choice of TSS can impact both RNA stability and potentially protein localization. They validate their results with qRT-PCR, RNA-seq and microscopy. They then perform a screen for transcription factors that may influence TSS usage for the gene *PKP1* and identify Tur1 as a potential regulator. A transcription factor that primarily regulates gene expression via TSS choice would represent an exciting finding in *Cryptococcus*, however a major challenge in making this claim is disentangling the impact of overall gene expression changes and/or RNA stability from TSS usage. In this case, the data presentation makes it difficult to evaluate the authors claim. This paper will likely be of broad interest to researchers in the fields of transcription and *Cryptococcus*. The conclusions would be strengthened by additional analyses and adjustments in data presentation.

1. Major points:

1. The presentation of short and long isoform usage as relative to ACT1 RNA make it difficult to evaluate whether it is primarily a TSS switch occurring, or largely a preferential stabilization/induction of one isoform. For example, in Figure 8B the IGV plots mapping TSS data appear consistent with a switch from the long isoform to short; however the RNA-seq data appear to show relatively unchanged usage of the longer isoform but a large increase in RNA from the shorter isoform and coding regions in stationary phase. This does not seem to be the conclusion from the accompanying qRT-PCR data so it is unclear how the overall impact on gene expression levels are being accounted for (this could be the result of changing axis height in the IGV data, but as the values are not indicated it is difficult to tell). Conclusions from all of the IGV/qRT-PCR data would be strengthened by including both the relative percent usage of each TSS per condition as well as including more information about the total read coverage.

2. The authors raise the possibility that *tur1* deletion causes widespread changes in transcription factor expression in exponential vs. stationary phase, but do not explore the implied possibility that some of the aTSS changes in the *tur1* mutant may therefore be indirect. It would strengthen their findings to show that Tur1 directly binds to one or more of the loci exhibiting changes, especially if the levels or localization of binding changes in exponential vs. stationary phase.

3. The localization data in Figure 6B are difficult to interpret and not entirely convincing as a result. Further quantification of re-localization would help, as would additional tagged positive and negative controls where (1) re-localization is known or (2) not expected.

Minor points

1. It would be helpful to view the location of the primers used in the qRT-PCR data in a schematic accompanying the data in the main text figure

2. In the sentence "In the tur1Δ mutant strain, this slowdown of the metabolism is less marked than in the WT strain, and we measured only a 1.5-fold reduction of the total number of RNA molecules per genome and identified only 27% (n =1,814) of the genes downregulated by at least two-fold" the authors likely mean to refer to figure 10A and 10B rather than figure 9A and 9B.

3. A few very minor grammatical suggestions:
"*using a RT-qPCR assay with primers specific of* ***[to]*** *the long or both the long and the short isoforms identified at the PKP1 gene"*

*"PKP1 transcription can start at two altTSS clusters with different expression pattern****[s]*** *in exponential and stationary phase"*

*"This mutant strain display****[ed]*** *no growth defect is mutated at the locus"*
