# Supplementary material for: Alternative TSS use is widespread in Cryptococcus fungi in response to environmental cues and regulated genome-wide by the transcription factor Tur1
Source: PLoS Biol. 2024 Jul 25;22(7):e3002724. doi: 10.1371/journal.pbio.3002724 (PMC11302930; doi:10.1371/journal.pbio.3002724)
Supplement: Attachment — Submitted filename: response to reviewers.docx [file pbio.3002724.s023.docx]

**Reviewer #1**

The authors performed genome-wide analysis for transcriptional start sites in two related Cryptococcus species. This study builds upon a recent work by the same group in which they mapped RNA transcripts versus the associated proteins, demonstrating a large number of upstream ORFs associated with inhibition of translation. They also demonstrated specific examples in which alternative transcript lengths resulted in altered protein localization, often by removing mitochondrial localization sequences.

In the current paper, the authors systematically and comprehensively define transcription start sites (TSS) for these Cryptococcus species, identifying thousands of alternative TSS. They further categorized TSS as occurring in the region prior to annotated ATGs, or further downstream, thus altering protein sequence. They were also able to identify condition-dependent alterations in TSS usage, providing strong evidence that this is a major means of protein regulation in this pathogenic fungus. Similar to their prior study, but on a much larger scale, they showed that some of these protein sequence changes resulted in altered localization.

Some of the general themes that they explore and define are consistent with paradigms from other species (e.g. S pombe). These include the observation that the present of TATA sequences in promoters is correlated with more dynamic, condition-specific, transcriptional regulation. However, the rigor with which these observations were defined is important to emphasize. Moreover, the relevance of these studies to host-pathogen interaction offers a very unique position for this paper relevant to other literature on this topic.

The logic of the experimental strategy is very clear, and the analysis is very rigorous. Data have been made available in accessible databases.

Minor issues.

1)	The presentation is mostly very clear. In one instance (Fig 6), however, the resolution of the protein localization was not completely evident by the provided images.

**We owe all three reviewers an apology as we realized that the submitted material was poorly resolved. The images provided in the first version of the manuscript were not clear enough, and the conversion of the file to pdf made them worse.**

**In the new version of the manuscript, we have performed new microscopic analyses using a better microscope. The new images shown in the new Figure 5B (formerly 6B) confirmed the differential localization of the protein Mae102 between exponential and stationary phase. This new material also confirmed that (1) the localization of Mae102 to mitochondrial-like particles is dependent on the presence of a mitochondrial targeting motif, as expected, and (2) the subcellular localization of Mae102 is regulated by Tur1. Actually, *TUR1* deletion impaired subcellular localization of the Mae102 protein in both growth phases in agreement with the altered alternative TSS usage pattern. In this new version of the manuscript, we have also quantified the number of cells showing nuclear localization of Mea102 in exponential phase in the different strains. These figures, shown as a percentage in the new Figure 5B, confirm the effect of both growth phase and Tur1 on Mae102 localization. The images used for this calculation as well as the corresponding raw numbers are shown in the new Figure S5.**

2)	The authors do not adequately explain the motifs identified by the MEME algorithm in the Figure 1C. How is this informative for the larger story? This seems to be an opportunity that is missed.

**We realized that these motifs were actually not discussed in the text. The main reason for this is that we have no idea what role they play here. We have added a sentence to explain this.**

***“As said above, we identified five additional enriched motifs proximal to the Cryptococcus TSS. Some of these motifs like “AAYKCCG” were more commonly associated with broad TSS some other as “GGRNG” were more commonly associated the with sharp TSS. Yet, their functionally remains to be studied.*** “

3)	Is the phrase "TATA-box containing genes" accurate since the sequence typically resides outside the ORF?

**In our view, genes include both the coding and regulatory regions, so the TATA box can be considered to be within the genes. We have kept "TATA-box containing genes" in the manuscript as this wording has been used in several papers before.**

4)	The authors could better describe how they developed their parameters for a TSS cluster to be "in close proximity" to the annotated ATG versus "far downstream". Is associated Fig 3 necessary?

**We agree with the reviewer and have deleted Figure 3. The explanation for each category of TSS is developed within the appropriate paragraph.**

5)	There are some instance of excessive descriptives, including e.g, "spectacular effect" (line 467); "… suggest a fantastic regulatory …" (line 588).

**We changed “spectacular” in “striking” and fantastic in “remarkable”**

6)	Line 535. "… no growth defect of the tur1 strain …"

**ok**

7)	Line 596. "… examples exist in model yeasts …"

**ok**

Reviewer #2

The authors characterise TSS usage in two species of pathogenic Cryptococcus, and define different TSS cluster characteristics (sharp and broad). They then look at differential TSS usage between different environmental conditions, characterising the effects based on the location of the aTSS with respect to the protein's annotated translation start codon. They describe an example gene where the subcellular localisation is supposedly altered by aTSS usage (resulting in the inclusion or exclusion of a localisation signal). They next look at the molecular mechanisms underpinning TSS usage and identify a TF (Tur1) which regulates downstream TSS usage in the stationary phase. They characterise the phenotype of Tur1 mutant strains.

Overall the manuscript is quite descriptive, often highlighting examples without further in detail characterization. Also the manuscript lacks some level of coherency. Terms are often poorly defined or not defined at all (e.g. alternative TSS). Moreover, often descriptions of how cut-offs are made are not included in main text making it difficult to appreciate the findings.

Specific main comments:

- The title of the manuscript does not really match the manuscript as only the last couple of figures are on Tur1, and Tur1 has an effect on subset of TSSs but not genome wide.

We changed the tittle in

**“Alternative Transcription Start Site usage in Cryptococcus”**

- It was not clear to me what the definition of alternative TSS entails. Based on reading the text it seems all genes that have 2 TSSs or more? It would be good to give a clear definition what it is alternative to. The literature often discriminates between main vs alternative TSS usage or dominant TSS vs weak TSS. In figure 4BC which of the TSSs is the alternative one?

**We thank the reviewer for this comment on the semantics of alternative TSS. We actually had a definition in the Materials and methods. This definition has now been moved to the main text to clarify our point.**

- Figure 6 the authors attempt to demonstrate a difference in localization. However this was difficult to assess. The images are of poor quality, and no merge was shown. Also no quantifications were shown, and no mitotracker for exp was shown.

**See response to reviewer 1**

- Tur1 effect on TSS usage looks interesting. However, it was not clear to me whether this was direct or indirect effect on TSS usage. Given that Tur1 also has a phenotype indirect effects are plausible. To discriminate between direct and indirect at least ChIP-seq of Tur1 is needed.

**This question is of great interest. To prepare the new version of this manuscript, we first performed ChiIP-seq analyses using a strain in which a 2xFlag::TUR1 fusion allele is expressed under the control of the ACT1 promoter. As described in this new version of the manuscript, analysis of our data confirmed that Tur1 can bind DNA and that a large proportion of the loci carrying genes regulated by Tur1 are also bound by this TF. We also identified genes regulated by Tur1 that are regulated by alternative TSSs. However, as indicated in this new version of the manuscript, the pattern of binding regulation is difficult to interpret in the light of aTSS usage. However, our ChIP-Seq experiments were performed using a strain in which the fusion protein is overexpressed. We reasoned that this overexpression might have masked informative regulation of Tur1 binding during the transition between exponential and stationary phase. We therefore performed DamID-Seq using a strain expressing a Dam-TUR1 allele under the control of the native TUR1 promoter. We identified a large number of sites where the methylation of adenine residues was regulated by growth phase, suggesting regulated Tur1 binding. However, again, the pattern of adenine residue methylation did not provide a simple model for Tur1-dependent regulation of aTSS usage.**

minor comments.

- manuscript would benefit from thorough read through spelling and acronym errors.

**We modified the text and corrected few mistakes.**

- proper definitions of terms used. E.g. Figure 3 what are the cut-off used.

**We deleted this figure**

Reviewer #3:

S The manuscript by Vi DANG et al. titled “Tur1 regulates alternative TSS usage in *Cryptococcus”* describes a new analysis of TSS data in *Cryptococcus* along with follow up experiments implicating the transcription factor Tur1 as a master regulator of aTSS. They demonstrate that many genes in *Cryptococcus* exhibit aTSS under different growth conditions, and that the choice of TSS can impact both RNA stability and potentially protein localization. They validate their results with qRT-PCR, RNA-seq and microscopy. They then perform a screen for transcription factors that may influence TSS usage for the gene *PKP1* and identify Tur1 as a potential regulator. A transcription factor that primarily regulates gene expression via TSS choice would represent an exciting finding in *Cryptococcus*, however a major challenge in making this claim is disentangling the impact of overall gene expression changes and/or RNA stability from TSS usage. In this case, the data presentation makes it difficult to evaluate the authors claim. This paper will likely be of broad interest to researchers in the fields of transcription and *Cryptococcus*. The conclusions would be strengthened by additional analyses and adjustments in data presentation.

Major points:

1. The presentation of short and long isoform usage as relative to ACT1 RNA make it difficult to evaluate whether it is primarily a TSS switch occurring, or largely a preferential stabilization/induction of one isoform. For example, in Figure 8B the IGV plots mapping TSS data appear consistent with a switch from the long isoform to short; however the RNA-seq data appear to show relatively unchanged usage of the longer isoform but a large increase in RNA from the shorter isoform and coding regions in stationary phase. This does not seem to be the conclusion from the accompanying qRT-PCR data so it is unclear how the overall impact on gene expression levels are being accounted for (this could be the result of changing axis height in the IGV data, but as the values are not indicated it is difficult to tell). Conclusions from all of the IGV/qRT-PCR data would be strengthened by including both the relative percent usage of each TSS per condition as well as including more information about the total read coverage.

**We thank the reviewer for this comment. We realized that the legend (short vs long) of the qPCR has been inverted in all figures of this type. We corrected this error and included the percentage of aTSS usage on the IGV data.**

2. The authors raise the possibility that *tur1* deletion causes widespread changes in transcription factor expression in exponential vs. stationary phase, but do not explore the implied possibility that some of the aTSS changes in the *tur1* mutant may therefore be indirect. It would strengthen their findings to show that Tur1 directly binds to one or more of the loci exhibiting changes, especially if the levels or localization of binding changes in exponential vs. stationary phase.

**See response to reviewer 2**

3. The localization data in Figure 6B are difficult to interpret and not entirely convincing as a result. Further quantification of re-localization would help, as would additional tagged positive and negative controls where (1) re-localization is known or (2) not expected.

**See response to reviewer 1**

Minor points

1. It would be helpful to view the location of the primers used in the qRT-PCR data in a schematic accompanying the data in the main text figure

**We included this information**

2. In the sentence “In the tur1Δ mutant strain, this slowdown of the metabolism is less marked than in the WT strain, and we measured only a 1.5-fold reduction of the total number of RNA molecules per genome and identified only 27% (n =1,814) of the genes downregulated by at least two-fold” the authors likely mean to refer to figure 10A and 10B rather than figure 9A and 9B.

**OK**

3. A few very minor grammatical suggestions:

“*using a RT-qPCR assay with primers specific of* ***[to]*** *the long or both the long and the short isoforms identified at the PKP1 gene”*

***OK***

*“PKP1 transcription can start at two altTSS clusters with different expression pattern****[s]*** *in exponential and stationary phase”*

***OK***

*“This mutant strain display****[ed]*** *no growth defect is mutated at the locus”*

***OK***
